# IL-12 and Related Cytokines: Function and Regulatory Implications in *Candida albicans* Infection

**DOI:** 10.1155/2011/686597

**Published:** 2010-11-01

**Authors:** Robert B. Ashman, Dipti Vijayan, Christine A. Wells

**Affiliations:** ^1^The School of Dentistry, University of Queensland School of Dentistry, 200 Turbot Street, Brisbane, QLD 4000, Australia; ^2^The National Centre for Adult Stem Cell Research, Eskitis Institute for Cell and Molecular Therapies, Griffith University, Brisbane, QLD 4111, Australia

## Abstract

IL-12 is a cytokine with links to both innate and adaptive immunity systems. In mice, its deletion leads to acute susceptibility to oral infection with the yeast *Candida albicans*, whereas such mice are resistant to systemic disease. However, it is an essential component of the adaptive response that leads to the generation of Th1-type cytokine responses and protection against disseminated disease. This paper presents an overview of the role of IL-12 in models of systemic and mucosal infection and the possible relationships between them.

## 1. Introduction

*Candida albicans* is a ubiquitous commensal yeast in the human oral cavity and gastrointestinal tract, as well as in the genital area of otherwise healthy females. While many individuals are asymptomatic, carriage increases the risks of contracting systemic infections [1, 2]. Virtually all clinical manifestations of disease tend to be associated with deficiency or dysfunction of the innate or adaptive arms of the immune system. Patients with defects in cell-mediated immunity tend to be susceptible to mucocutaneous, but not to disseminated, candidiasis, whereas systemic infections are more commonly associated with neutropenia or dysregulation of neutrophil function.

**Figure 1:**
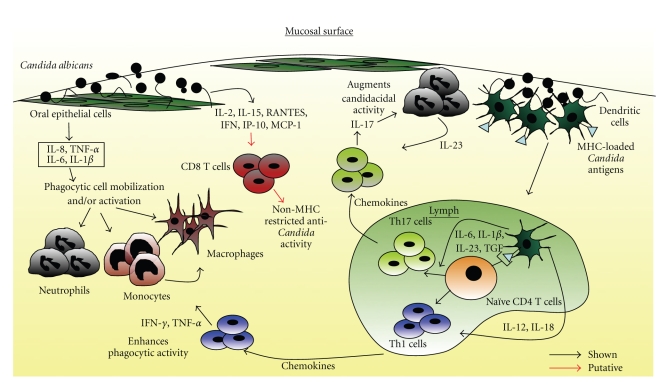
A schematic illustrating known and postulated pathways of response to infection with* Candida albicans.* Interaction with epithelial cells causes the release of cytokines and chemokines that recruit and activate inflammatory and immune cells, including phagocytes, antigen presenting cells (APCs), and T cells. Phagocytic cells engulf the invading fungus and kill via the respiratory burst and cytokine release, whereas APCs process the *Candida *antigens and migrate to the lymph nodes to present them, in the context of the MHC Class II molecule, to naive CD4 T cells, which are then activated and differentiate to either a Th1-type or a Th17-type cell. The dominant outcome (Th1 or Th17) is probably determined by the prevailing cytokine milieu. On reaching the infected site, Th1 effector cells release cytokines that orchestrate containment of infection to the mucosal surfaces and prevent dissemination. Th17 cells release IL-17, thereby enhancing the candidacidal activity of neutrophils. Thus, both innate and adaptive components of the immune system work cooperatively to provide an effective defence against the invading yeast.

Candidiasis manifests as a symptom of a number of clinical conditions that have been extensively discussed elsewhere [3]. Our understanding of host responses to this pathogen remains incomplete; however, emerging data, depicted in Figure 1, indicate that the production of key cytokines, such as IL-12 and IL-23, by the innate immune system is essential in the recruitment of appropriate adaptive responses in mucocutaneous disease.

## 2. The IL-12 Family: Structure and Function

IL-12 is a cytokine produced predominantly by innate immune cells, including epithelial cells, dendritic cells, and macrophages, although it is often referred to as a B cell cytokine. It acts as a multimer linked by disulphide bonds—most commonly as the p70 heterodimer consisting of p35 and p40 subunits, although the p40 homodimer, sometimes referred to as IL-12p80, is the dominant effector molecule produced by epithelial cells. All forms of IL-12 bind to the IL-12 receptor, which is also a heterodimer of IL-12Rb1 and RbII subunits that are expressed predominantly on T cells and on natural killer (NK) cells. IL-12 plays a critical role in inducing Th1 responses [4], which in turn leads to the production of a number of cytotoxic cytokines, as well as interferon-gamma (IFN-*γ*) by T cells. IFN-*γ* is itself a major determinant of Th1 maturation and a dominant modifier of phagocyte phenotypes. 

The p40 subunit of IL-12 is shared with IL-23, a related cytokine with profound differences in the cellular outcomes that it elicits. IL-23 is a heterodimer of IL-12p40 and IL-23p19 [5] and is also expressed by innate immune cells in response to *Candida *challenge. The IL-23 receptor complex also shares a subunit with the IL-12R, binding with high affinity to the IL-12Rb1 subunit but in a heterodimer with a novel IL-23R subunit. The ability of human T cells or mouse bone-marrow derived macrophages to respond to IL-12 or IL-23 is dependent on differential expression of the IL-12Rb2 or IL-23R subunits, respectively [6].

IL-23 and IL-12 appear to have important and complementary roles in the induction of  T-cell responses to *Candida*. Most significantly, IL-23 is essential for polarisation of the Th17 response [7], which has an increasingly important role in the host response to *Candida* infection [8]. It also increases local activity of the matrix metalloproteinase, MMP-9, increases angiogenesis, and reduces CD8^+^ T-cell infiltration [9]. 

Because of the structural similarities and shared receptor subunits, mouse knockout studies describing the effects of IL-12 may in fact be reporting compound phenotypes. In particular, IL-12p40-null animals will be null for three molecules: IL-12p70, IL-12p80, and IL-23. The role of IL-12p80, which is capable of binding both the IL-12R and IL-23R via the IL-12Rb1 subunit, also generates a phenotype that remains underexplored in mouse models of candidiasis, or in human clinical disease, because of this structural overlap.

## 3. Dissecting the Role of IL-12/IL-23 in Candidiasis Using Experimental Mouse Models

*C. albicans* appears to be unique in its ability to infect a variety of tissues and organs, including kidney, brain, oral and vaginal mucosa, skin, and gastrointestinal tract. This has led to the establishment of a plethora of animal models [10], many with quite disparate objectives, and although each offers many opportunities for advancement of knowledge in particular situations, the very diversity of these models has made it exceptionally difficult to derive a comprehensive understanding of the host/pathogen interaction. Many of the issues have been discussed previously, by the present authors [11], and others [12]. Given the central role of the IL-12 family in the linkage between innate and adaptive immunity, the purpose of this paper is to identify commonalities and discrepancies between models of various manifestations of infection and perhaps thus identify crucial issues, the resolution of which would facilitate their integration into an overall concept of the disease process.

### 3.1. Systemic Candidiasis

The importance of innate immunity was first demonstrated by the observation that a deficiency in the fifth component of complement markedly increased susceptibility to lethal infection with *C. albicans* in mice [13], although this did not affect the development of specific immune responses in mice that survived [14]. However, neither the severity nor the duration of kidney infection in C5-deficient mice was influenced by depletion of T cells [15]. 

Indeed, T-cell-deficient “nude” [16] and SCID [17] mice were shown to be no more susceptible than controls to systemic challenge, and this was confirmed by studies in mice lacking the *α*/*β* chains of the T-cell receptor [18]. These results demonstrated that innate immune responses were effective in clearance of the infection, even in the absence of T-cell-derived cytokines. Furthermore, gene profiling studies of human monocytes [19] have found a marked enhancement of proinflammatory cytokines during the first 6 hours after *ex vivo* exposure to *C. albicans*, supporting a dominant role for innate immunity in the early response against the yeast.

In contrast, there is now a substantial body of literature on the role of T cells in the development of protective immune responses following systemic challenge. The earliest of these studies used various combinations of yeasts (attenuated and virulent), and mouse strains (BALB/c and DBA/2), to produce healing infections that resolved with the development of resistance to reinfection, or nonhealing infections that led to chronic disease and death [12]. Healing combinations showed the development of a Th1 cytokine profile by CD4^+^ spleen cells *in vitro*, whereas a Th2 profile was demonstrated in lymphocytes from animals with chronic disease. In this context, it is worth noting that binding of C5a to its receptor on antigen-presenting cells upregulates IL-12p70, which in turn contributes to IFN-*γ* directed T-cell differentiation toward a Th1 phenotype [20]. Consequently, the C5-deficient DBA/2, that typically displayed susceptibility to rechallenge [21], may have a propensity to develop Th2-type responses, independent of the nature of the *Candida* challenge. 

In these models, IL-12 was readily demonstrable in healing infections, whereas the archetypal Th2 cytokine, IL-4, was found only in animals with progressive infections [21]. Neutralization of IFN-*γ* prevented the development of protective Th1 responses [22], whereas neutralization of IL-4 reduced mortality, with the induction of a Th1 cytokine profile followed by the development of protective immunity [23]. In sublethal infection, neutralization of IL-12 ablated the development of resistance, but administration of exogenous IL-12 did not result in therapeutic activity [24]. Suppression of a Th2 phenotype—induced in these studies by treatment of the mice with antibodies against IL-4 or IL-10—enabled them to overcome the infection and led to the development of Th1 cytokine profiles [25]. Modulation of putative interactions between Th1/Th2 cytokines in the context of *Candida* infections has been postulated as having therapeutic potential [25], although recent studies have demonstrated increasing complexity in such responses.

The effects of IL-12 in these models are presumably delivered through induction of IFN-*γ* as mice in which the IFN-*γ* receptor had been deleted failed to mount protective Th1-mediated acquired immunity in response to a live vaccine strain of the yeast [26]. The impaired Th1-mediated resistance correlated with defective IL-12 responsiveness, but IL-12 production was unimpaired. Although inhibition of Th2 cytokines IL-4 and IL-10 is associated with a reciprocal polarization towards Th1 responses, germline loss of IL-4 did not recapitulate these results. IL-4-deficient mice were more resistant than wild-type animals early after systemic challenge [27], but eventually succumbed. A failure to develop Th1-mediated responses in these mice was associated with defective IFN-*γ* and IL-12 production, but they retained responsiveness to IL-12. Administration of exogenous IL-4 in the later stages of infection upregulated Th1-type responses and improved outcomes. These data do not readily reconcile a role for IL-4 in resolving systemic disease; however, they do demonstrate the importance of being able to mount an effective Th1 response, which is primarily mediated via IL-12/IFN-*γ*. 

Although the above makes an impressive case for the primary role of T helper cells in generating protective responses against *C. albicans* infection, tissue susceptibility is known to be determined by the genetic background of the mouse [28], and this is associated with different patterns of immune reactivity in the host [29, 30]. Consequently, it might be expected that some of the disparities observed in various experimental systems could be attributed to the background on which the mice were bred (Table 1). Lavigne and colleagues [31] examined the effects of IL-12 and IFN-*γ* in the response of C57BL/6 mice to systemic infection. IL-12 treatment, either alone or in combination, resulted in the generation of a Th1-like cytokine profile, with little evidence of IL-4, but paradoxically, treatment with IL-12 increased the severity of the disease. Antibody-mediated neutralisation of IFN-*γ* countered the deleterious effects of IL-12 administration, suggesting that the induced lethality was mediated by IFN-*γ*. This conclusion, however, was not consistent with results using IFN-*γ* knockout mice, which varied depending on the genetic background. If the knockout was on the BALB/c background, there was no effect on systemic or gastric candidiasis [32], whereas Balish [33] found that C57BL/6 × 129 IFN^−/−^ mice were more susceptible to gastric, anorectal, and acute systemic candidiasis than were immunocompetent controls IFN^−/−^ mice bred on the C57BL/6 background also showed increased mortality following systemic challenge [34], but this did not reflect an increased fungal burden in the tissues. IL-18 and IL-12 are strong stimulators of IFN-*γ* production, and in a separate study [35], IL-18^−/−^, but not IL-12^−/−^, mice bred on the C57BL/6 background displayed a similar increased mortality following systemic challenge with *C. albicans*. This was attributed to a marked reduction in production of IFN-*γ* and a concomitant decrease in MIP-2 (macrophage inflammatory protein-2) in the IL-18^−/−^ mice, and in KC (keratinocyte chemoattractant) in both, resulting in a reduced influx of monocytes at the site of infection.

Mortality following *C. albicans* infection in outbred Crl:CF-1 mice was associated with induction of IL-10 [39]. IL-12 and IFN-*γ* were not detected, and production of tumour necrosis factor-alpha (TNF-*α*) was delayed. A strong effect of the genetic background of the mouse was also observed after administration of CpG oligodeoxynucleotide, which has been shown to induce IL-12. The effect was deleterious in BALB/c [40], whereas in C57BL/6, it conferred protection that correlated with the early expression of IL-12 mRNA in the kidneys [41], and an increase in the levels of IL-12 in the serum. The protective response could not be induced in (B6; 129SF2/J) TNF-*α* knockout mice [41], indicating that IL-12 was induced via a TNF-*α*-dependent pathway. The relationship, however, is not straightforward, as TNF/lymphotoxin (LT)-*α* double-knockout mice on the same background were found to be more susceptible to infection, in spite of the production of IL-12 [42].

Some of these apparent conflicts may be attributable to the morphological plasticity of the growth forms of the organism and the relative mass of yeasts versus hyphae during the initial phases of the host response. *In vitro*, ingestion of yeasts activated dendritic cells for IL-12 production and priming of Th1 cells, whereas ingestion of hyphae inhibited IL-12 and Th1 priming and induced IL-4 production [43]. Human monocytes cultured with granulocyte-macrophage colony-stimulating factor and IL-4 after phagocytosis of *Candida* yeasts did not differentiate into dendritic cells yet secreted high levels of TNF-*α* and IL-10, but not IL-12 [44]. Monocytes that had phagocytosed germinating forms differentiated into mature dendritic cells but were unable to produce IL-12. These cells were able to prime naive T cells but not to induce their functional polarization into effector cells. However, early stages of the dendritic cell response tended to be dominated by the secretion of pro-inflammatory and inflammatory mediators, such as IL-8 and TNF-*α*, although blastoconidia induced markedly lower cytokine levels than filamentous forms [45].

Inflammatory responses are a dominant feature of the early response to *C. albicans*, but these must be under precise control in order to avoid excessive tissue damage. Dendritic cells also produce IL-23, which has been shown to regulate the production of IL-12 [46], thus providing a balance between host resistance and uncontrolled inflammation. Of interest is the role of IL-17A in mediating resistance to systemic infection [47], particularly as exposure of dendritic cells to hyphae rather than yeast forms has recently been shown to shift the host toward a Th17 rather than Th1 style of response [48].

The balance between IL-12 and IL-23 may be determined by the interactions between the various pattern recognition receptors and antigenic determinants of the yeast. Interaction through Dectin-1 induced the maturation of dendritic cells and the secretion of proinflammatory cytokines, including IL-6, TNF-*α*, and IL-23, but little IL-12 [49], whereas Dectin-2 was activated predominantly by the yeast form [50]. Activation through both Dectin-1 and Dectin-2 promoted the differentiation of Th17 cells, which appear to be essential for host resistance as IL-17A-deficient mice were highly susceptible to systemic infection [47, 50].

Although the experiments outlined above suggested that IL-12 played a central role in the development of protective immune responses against systemic candidiasis, IL-12p40 KO mice were found to be strikingly susceptible to oral infection, which persisted undiminished for several months [11], whereas recovery from systemic challenge was unaffected. Interestingly, Conti et al. [38] found that IL-12p35^−/−^ mice, which had impaired Th1 responses, demonstrated only low levels of oropharyngeal colonization and no overt disease. In contrast, Th17-deficient (IL-23p19^−/−^) and IL-17R-deficient (IL-17RA^−/−^) mice developed severe infections. As mice deficient in the Th17 cytokine IL-22 were only mildly susceptible, they concluded that the Th17 lineage, acting largely through IL-17, mediates the response to oral candidiasis via the actions of neutrophils and antimicrobial factors.

The issue is further complicated by the demonstration that germinating *C. albicans* fails to induce IL-12 p70 [51], an effect mediated by a soluble product of the germinating cells that acted by induction of phosphorylation of ERK44/42 MAPK [52]. Clearly, there are many levels of interaction between yeasts and host cells that can influence the evolution of both innate and adaptive immune responses.

### 3.2. Mucocutaneous Candidiasis

In contrast to their resistance to systemic infection, T-cell-deficient (nude) mice are acutely susceptible to oral infection [53]. Furthermore, mice in which the T-cell receptor *δ*- and *α*-chains had been genetically deleted were also highly susceptible to orogastric candidiasis [18] but remained resistant to acute systemic candidiasis. These observations strongly suggest that the predominant host response is different in the two types of infection. 

#### 3.2.1. Gastrointestinal Candidiasis

Both normal and T-cell-deficient mice develop orogastric candidiasis [54], but only T-cell-sufficient mice demonstrate *Candida*-specific lymphoproliferation and DTH responses that correlate with the clearance of *C. albicans* hyphae from mucosal surfaces. Depletion of CD4^+^ lymphocytes increased their susceptibility to *Candida* infection of the tongue and esophagus [55], but treatment with anti-IL-2 anti-IFN-*γ*, or both did not abrogate their resistance. B-cell knockout mice, which lack both functional B cells and antibodies, were as resistant to orogastric candidiasis as immunocompetent controls [56] a result consistent with a primary role for cell-mediated immunity in host protection against mucocutaneous candidiasis. The *γ*/*δ* T cells of the mucosa have also been implicated in the process of host defence [57]. 

Although Th1 responses are important for recovery from disseminated candidiasis, responses in gastrointestinal infection were also related to the virulence of the yeast used for challenge and the “healer” or “nonhealer” status of the mice. BALB/c mice were resistant to systemic challenge with the avirulent yeast and generated a protective Th1 cytokine response [58]. In contrast, systemic infection with the virulent isolate caused early mortality, whereas gastrointestinal colonisation with the same yeast resulted in the production of both Th1- and Th2-type cytokines by CD4^+^ cells from Peyer's patches and mesenteric lymph nodes, at a time when the yeasts were being cleared from the intestine [59]. Although C5-deficient DBA/2Cr mice developed Th2-type responses and fatal disseminated candidiasis after intravenous infection with the avirulent strain yeast [58], intragastric challenge with the virulent strain resulted in the induction of Th1-type cell-mediated immune responses and eventual clearance of the infection [60], demonstrating again the discrepancy in host responses following systemic or mucosal challenge.

Administration of soluble IL-4 receptor to mice with gastrointestinal candidiasis accelerated the clearance of the fungus from the stomach and stimulated Th1-associated resistance [61]. IL-12-deficient mice were found to be highly susceptible to primary gastrointestinal infection or reinfection, and showed elevated production of IL-4, as well as reduced production of IFN-*γ* [37]. Treatment of IL-12-deficient mice with exogenous IL-12 or IL-10 impaired IL-4 production, and increased resistance to infection, through a negative effect on the CTLA-4/B7-2 costimulatory pathway. 

Although mice in which the genes for particular cytokines had been deleted were expected to reveal relevant relationships, such studies have not been particularly illuminating. As noted above, the responses of KO mice appeared to be heavily influenced by the genetic background. Ablation of IL-10 increased resistance against both gastrointestinal [62] and systemic [63] candidiasis in mice bred on the C57BL/6 background, but deletion of the gene for IL-4 had no effect [34]. 

A previously unrecognized complication in the interpretation of cytokine profiles in these models comes from the observation that immunocompetent mouse strains may develop different cytokine profiles, even though they show a comparable susceptibility to infection. Germ-free C57BL/6 and BALB/c mice were equally susceptible to intestinal colonization with *C. albicans* and had similar fungal burdens in gastric tissues 4 weeks after oral challenge [64]; however, C57BL/6 mice responded with increased expression of TNF-*α*, IL-12, and the chemokines MIP-2 and KC, whereas a much more specific and attenuated response was observed in *Candida*-infected gastric tissues from BALB/c mice.

#### 3.2.2. Oral Candidiasis

 There is now strong evidence that CD4^+^ T cells are essential for clearance of primary infection from the oral cavity of mice, and these results are consistent with clinical observations. Both BALB/c and DBA/2 mice showed a comparable recruitment of CD4^+^ and CD8^+^ T lymphocytes into the mucosal tissue [65] as well as a substantial increase in intraepithelial CD4^+^ T cells [66] and an influx of *γ*/*δ* T cells [67] that correlated with a substantial decrease in the number of viable organisms recovered from the mucosal tissue [65]. 

However, mouse strain-specific effects were also observed, as there was significantly higher *Candida*-specific T-cell proliferation in BALB/c as compared to DBA/2 mice [67]. In the resistant BALB/c mice, rapid clearance of *C. albicans* from the oral mucosa was associated with an early increase in levels of IL-4, IL-12, and IFN-*γ* in cells from the cervical lymph nodes, whereas in DBA/2 mice, which cleared the infection more slowly, IL-4 mRNA was detected later, and the levels secreted were lower [67]. In BALB/c mice, monoclonal antibody neutralization of IL-4 increased the fungal burden and delayed clearance of the yeast. Thus, in contrast to results in systemically infected mice [23], IL-4 appeared to be an important mediator of protection in oral candidiasis. Unexpectedly, inhibition of IL-4 production in susceptible DBA/2 mice was accompanied by an increase in IFN-*γ* production [68], which tended to argue against a strict Th1/Th2 dichotomy as the sole determinant of resistance and susceptibility in oral candidiasis. 

The importance of the T-cell response was demonstrated by the increased levels of oral colonization in nude mice, that develop a severe infection that does not clear [53]. Established infections in nude mice could be cleared by the adoptive transfer of syngeneic CD4^+^ but not CD8^+^ lymphocytes [53]. Recovery was associated with the presence of IFN-*γ* and IL-12 in the cervical and submaxillary lymph nodes of the reconstituted mice. IL-12 and IFN-*γ* were the major cytokines produced by lymphocytes from the draining lymph nodes of animals recovering from oral infection [69], but levels of IL-4 and IL-10 did not show any association with recovery. Further studies of oral candidiasis in IFN-*γ*, IL-4, IL-10, and iNOS KO mice also failed to demonstrate any alteration in the severity or course of the disease [36]. In TNF-*α* KO mice, there was an early increase in the fungal burden in the oral cavity, but the duration of the infection was not different from controls, and TNF-*α* was the only cytokine that was detected specifically in infected oral mucosa [70].

This group [71] has also conducted gene-profiling studies of oral mucosa and draining lymph nodes following oral colonisation in wild-type and IL-12p40 knockout mice six days after challenge. They found an upregulation of soluble inflammatory markers and proteins involved in tissue remodelling, although at this time after infection, it could be expected that the infection would be beginning to be cleared and healing would commence. Conti et al. [38] similarly demonstrated upregulation of genes associated with inflammation, and specifically Th17 function, in the oral mucosa at 24 hours after infection. As depletion of neutrophils, or inactivation of macrophages/monocytes, increased the severity of infection [69], there appears to be a clear linkage between the induction of Th17 cells and the functional activities of neutrophils, and possibly other phagocytic cells, in clearance of the yeast. 

In oropharyngeal candidiasis, the mucosa represents the target area for infection, as well as the site that phagocytic and other effector cells must reach in order to combat the infection. The matrix metalloproteinases (MMP) are enzymes crucial for physiological homeostasis, as well as pathological chemotaxis of immune cells to target tissues [72]. Following exposure to *C. albicans*, engineered normal human oral mucosa showed laminin-5 and type IV collagen gene activation and protein secretion, as well as MMP-2 and MMP-9 gene activation, although only the latter was associated with an increase in active MMP-9 [73]. It was suggested that these effects might enable *C. albicans* to overcome the mechanical and biological defenses of the tissue and allow it to disseminate, although systemic dissemination following oro-pharyngeal infections in humans is uncommon.

In the present context, it is worth noting that both pro- and anti-inflammatory cytokines can modulate MMPs and their tissue inhibitors. IL-18 and IL-12 both separately and synergistically enhanced MMP-2, while TNF-*α* led to the elevation of MMP-9. All proinflammatory cytokines enhanced MT1-MMP expression, and IL-4 suppressed TNF-*α*-induced MMP-9 expression [74]. On the other hand, the inhibitory effect of IL-12 on MMP9 expression in activated peripheral blood mononuclear cells (PBMCs), as well as their ability to transmigrate across an extracellular matrix, was enhanced in the presence of endothelial cells, and conversely, stimulated PBMCs reduced the expression and the activity of MMP9 [75]. It is, therefore, possible that the presence of TNF-*α* in the oral mucosa in the early stages of infection [70] may, by stimulating production of MMP9, be instrumental in facilitating the passage of inflammatory cells into the oral mucosa, whereas later in the course of infection, IL-12 downregulates this activity.

## 4. Conclusion

A concept that would integrate the disparate responses to systemic and mucosal candidiasis remains elusive. IL-12, thought to be at the centre of such responses, has been reevaluated in the light of the functions of IL-23 and somewhat overshadowed in importance by the Th17 cells and their cytokines, whose pivotal role in oral candidiasis now seems well established. However, although the evidence indicates that IL-17 does play a role in the response against systemic infection, the precise role of T cells in this system is unclear, and inconsistencies and contradictions between the various models still remain. Although approaches such as gene profiling identify a plethora of candidate genes either up- or downregulated in host responses, validation of the biochemical pathways involved will still be required, and confirmation in animal models remains the gold standard.

## Figures and Tables

**Table 1:** Summary of effects of deletion of selected cytokine genes on susceptibility to systemic and mucosal candidiasis in mice.

Gene deleted	Background	Oral/Gastrointestinal response	Systemic response	Reference
IFN-*γ*	BALB/c	No effect	No effect	[32]
IFN-*γ*	C57BL/6 × 129	Susceptible	Susceptible	[33]
IFN-*γ*	C57BL/6	ND*	Susceptible	[34]
IL-4	BALB/c	ND	No effect (early)	[27]
IL-4	BALB/c	No effect	No effect	[36]
IL-18	C57BL/6	ND	Susceptible	[35]
IL-12 (p40)	BALB/c	Susceptible	No effect	[37]
IL-12 (p40)	C57BL/6	ND	No effect	[35]
IL-12 (p40)	C57BL/6	Susceptible	No effect	[11]
IL-12 (p35)	C57BL/6	No effect	ND	[38]
IL-23 (p19)	C57BL/6	Susceptible	ND	[38]

*ND, not done.
